# Involvement of Fatty Acid Amide Hydrolase and Fatty Acid Binding Protein 5 in the Uptake of Anandamide by Cell Lines with Different Levels of Fatty Acid Amide Hydrolase Expression: A Pharmacological Study

**DOI:** 10.1371/journal.pone.0103479

**Published:** 2014-07-31

**Authors:** Emmelie Björklund, Anders Blomqvist, Joel Hedlin, Emma Persson, Christopher J. Fowler

**Affiliations:** 1 Department of Pharmacology and Clinical Neuroscience, Umeå University, Umeå, Sweden; 2 Department of Radiation Sciences, Umeå University, Umeå, Sweden; Sapienza University of Rome, Italy

## Abstract

**Background:**

The endocannabinoid ligand anandamide (AEA) is removed from the extracellular space by a process of cellular uptake followed by metabolism. In many cells, such as the RBL-2H3 cell line, inhibition of FAAH activity reduces the observed uptake, indicating that the enzyme regulates uptake by controlling the intra- : extracellular AEA concentration gradient. However, in other FAAH-expressing cells, no such effect is seen. It is not clear, however, whether these differences are methodological in nature or due to properties of the cells themselves. In consequence, we have reinvestigated the role of FAAH in gating the uptake of AEA.

**Methodology/Principal Findings:**

The effects of FAAH inhibition upon AEA uptake were investigated in four cell lines: AT1 rat prostate cancer, RBL-2H3 rat basophilic leukaemia, rat C6 glioma and mouse P19 embryonic carcinoma cells. Semi-quantitative PCR for the cells and for a rat brain lysate confirmed the expression of FAAH. No obvious expression of a transcript with the expected molecular weight of FLAT was seen. FAAH expression differed between cells, but all four could accumulate AEA in a manner inhibitable by the selective FAAH inhibitor URB597. However, there was a difference in the sensitivities seen in the reduction of uptake for a given degree of FAAH inhibition produced by a reversible FAAH inhibitor, with C6 cells being more sensitive than RBL-2H3 cells, despite rather similar expression levels and activities of FAAH. The four cell lines all expressed FABP5, and AEA uptake was reduced in the presence of the FABP5 inhibitor SB-FI-26, suggesting that the different sensitivities to FAAH inhibition for C6 and RBL2H3 cells is not due to differences at the level of FABP-5.

**Conclusions/Significance:**

When assayed using the same methodology, different FAAH-expressing cells display different sensitivities of uptake to FAAH inhibition.

## Introduction

The endogenous cannabinoid ligand anandamide (arachidonoylethanolamide, AEA) is produced “on demand” [1] and removed from the synaptic cleft by a process of cellular uptake followed by metabolism, primarily by the intra-cellular enzyme fatty acid amide hydrolase (FAAH) [2]. The process of the cellular clearance has been widely discussed in the literature (for review, see [3]) and several intracellular AEA transporters such as fatty acid binding protein 5 and 7, heat shock protein 70 and albumin have been proposed [4], [5]. An FAAH-like AEA transporter (FLAT) has also been proposed as an intracellular carrier protein [6], although this has been disputed [7].

In 2001, two papers were published linking the uptake of AEA to its FAAH-catalysed breakdown. Day et al. [8] reported that transfection of HeLa cells with FAAH increased the observed rate of AEA uptake, and that inhibition of the enzyme in RBL-2H3 basophilic leukaemia cells reduced the uptake. Deutsch et al. [9] found that uptake was reduced (but not completely blocked) in FAAH-containing C6 glioma and N18 neuroblastoma cells following inhibition of the activity of this enzyme by the admittedly non-specific compounds methylarachidonoylfluorophosphonate and phenylmethylsulfonyl fluoride, whereas these compounds were without effect on the uptake of Hep2 laryngeal carcinoma cells, which lack FAAH. The authors suggested that FAAH gated the uptake of AEA by hydrolysing the intracellularly accumulated compound, and thereby preserving its extra- : intracellular gradient [8], [9]. Selective FAAH inhibitors such as URB597 [10] are now available, and a role for FAAH in regulating the uptake of AEA in several cells has been demonstrated using this compound (see e.g. [11], [12]) In a recent study, it was reported that AEA applied to the outside of synthetic lipid vesicles could be hydrolysed if FAAH was attached to the inside of the vesicles, and that the rate of hydrolysis was increased if cholesterol was added to the membrane, leading the authors to argue that the endocannabinoid can be internalised and presented to FAAH without the absolute requirement for membrane translocating proteins [13], [14].

Although these and other studies clearly argue in favour of a role of FAAH in regulating AEA uptake, other studies have reported the opposite, namely that the presence of FAAH in a cell is not a determinant of the uptake. Thus, almost complete inhibition of FAAH in cortical astrocytes by 25 µM (E)-6-(bromomethylene)tetrahydro-3-(1-naphthalenyl)-2H-pyran-2-one does not affect the uptake of AEA into these cells, and a similar result was seen with 15 µM linoleyl trifluoromethyl ketone [15]. AEA can also be accumulated by synaptosomes from FAAH^−/−^ mice [16]–[18]. Conversely, AEA uptake into human astrocytoma cells and cultured rat cortical neurones can be completely blocked by AM1172, a compound that is a weak FAAH inhibitor [16] although a subsequent study argued that this compound did not affect the uptake of AEA into RBL-2H3 cells when a short (25 second) incubation time was used [19].

From the above discussion, there are clearly disagreements in the literature concerning the degree to which FAAH contributes to the regulation of cellular AEA uptake. While it is possible that these differences are due to cellular diversity, it is also possible that methodological differences can contribute to the observed differences. One way of distinguishing between these possibilities is to use a standardised method to compare the sensitivity of different FAAH-containing cells to inhibition of the enzyme. This has been undertaken in the present study.

## Methods

### Compounds

Anandamide [arachidonoyl 5,6,8,9,11,12,14,15-^3^H] (specific activity 200 Ci/mmol; for the uptake experiments) and anandamide [ethanolamine-1^3^H] (specific activity 60 Ci/mmol; for the FAAH assay) was obtained from American Radiolabeled Chemicals Inc. (St. Louis, MO, USA). URB597 (3′-carbamoyl-biphenyl-3-yl-cyclohexyl-carbamate) was obtained from the Cayman Chemical Company (Ann Arbor, MI, USA), Compound 33 [20] was provided by Dr. Valentina Onnis, University of Cagliari, Italy. SB-FI-26 was a kind gift from Drs. Dale Deutsch and Iwao Ojima, Stony Brook University, NY, USA.

### Cell cultures

All cell types used were grown in 75 cm^2^ culturing flasks at 37°C with 5% CO_2_ in humidified atmospheric pressure. Passage of cells occurred approximately twice a week and medium was changed every other day. Rat basophilic leukaemia RBL-2H3 cells (passage range 13–72), obtained from the American Type Culture Collection (Manassas, VA, USA) were cultured in minimum essential medium with Earle’s Salts (MEM), foetal bovine serum (FBS) (15%), penicillin 100 U ml^−1^ + streptomycin 100 µg ml^−1^ (PEST). Rat prostate cancer AT1 cells (passages 34–58) were obtained from Professor Anders Bergh, Department of Medical Biosciences, Umeå University. The cells (sometimes termed R3327 AT1, and originating from the inventor, [21]) were cultured in RPMI 1640 medium, with 10% FBS, 25 nM dexamethasone, 2 mM l-glutamine and PEST. C6 rat glioma cells (passage range 12–38), obtained from the European Collection of Cell Cultures, (Porton Down, UK) were cultured in F-10 Ham supplemented with 10% FBS and PEST. P19 mouse embryonic carcinoma cells (passage 18–33, European Collection of Cell Cultures, Porton Down, UK), were cultured in MEM alpha 22571 with 10% FBS, 1% non-essential amino acids and PEST.

### AEA uptake assay

The method of Rakhshan et al. [22] with minor modifications [23] was used. Cells (RBL-2H3, AT1, C6 and P19) were plated at a density of 2×10^5^ cells per well in 24-well culture plates and incubated overnight at 37°C in an atmosphere of 5% CO_2_. One plate of wells containing only medium was also incubated and subsequently used to determine the non-specific retention of [^3^H]AEA to the plates. After incubation, cells were washed once with Krebs-Ringer Hepes (KRH)-buffer (120 mM NaCl, 4.7 mM KCl, 2.2 mM CaCl_2_, 10 mM 4-(2-hydroxyethyl)-1-piperazineethane-sulfonic acid (HEPES), 0.12 mM KH_2_PO_4_, 0.12 mM MgSO_4_ in milliQ deionised water, pH 7.4) containing 1% bovine serum albumin (BSA) and once with KRH- buffer alone. Cells were preincubated with KRH containing 0.1% fatty acid-free BSA and test compounds or vehicle control (dimethyl sulfoxide for URB597 and SB- FI-26, ethanol for Compound 33; vehicle concentrations are indicated in the figure legends) for 10 minutes at 37°C. [^3^H-arachidonoyl]AEA (unless otherwise stated; 50 µl, final concentration of 100 nM, in KRH-buffer containing 0.1% fatty acid-free BSA) was added to give a final volume of 400 µl, and the wells were incubated for 4 minutes at 37°C, unless otherwise stated. The plates were put on ice before washing three times with 500 µl of KRH-buffer containing 1% BSA. The buffer was removed, 0.2 M NaOH (500 µl) was added and the plate incubated at 75°C for 15 minutes to solubilise the cells. Aliquots of 300 µl were transferred to scintillation vials and the tritium content was determined by liquid scintillation spectroscopy with quench correction.

### FAAH assay

The FAAH assay was carried out essentially as described by Boldrup et al. [24] using lysates from C6 and RBL-2H3 cells. Briefly, 5 µg protein diluted with 10 mM Tris-HCl, 1 mM EDTA pH 7.4, test compound (or vehicle control, final assay concentration 1%) and [^3^H-ethanolamine]AEA in 10 mM Tris-HCl, 1 mM EDTA, pH 7.4, containing 1% w/v fatty acid-free BSA, final substrate concentration of 0.5 µM were incubated for 10 min at 37°C. Blanks contained buffer instead of membrane preparation. Reactions were stopped by placing the tubes on ice and adding 80 µL activated charcoal mixed in 320 µL of 0.5 M HCl. Samples were mixed and left at room temperature for about 30 min prior to centrifugation at 2500 rpm for 10 min. Aliquots (200 µL) of the supernatants were analysed for tritium content by liquid scintillation spectroscopy with quench correction. Ethical permission for the animal samples was obtained from the local animal research ethical committee (Umeå Ethical Committee for Animal Research, Umeå, Sweden).

For the experiments in intact cells, the assay described above for AEA uptake was used with two changes: a) [^3^H-ethanolamine]AEA rather than [arachidonoyl 5,6,8,9,11,12,14,15-^3^H]AEA was used, and b) the reaction was stopped by addition of 120 µL activated charcoal mixed in 480 µL of 0.5 M HCl [25]. Aliquots (600 µL) were pipetted into glass tubes, which were then centrifuged and aliquots (200 µL) of the supernatants were analysed for tritium content by liquid scintillation spectroscopy with quench correction. Blanks were wells alone. An initial experiment using C6 cells indicated that when an initial number (i.e. added to wells and incubated overnight) of 10^5^ cells/well were used, the amount of substrate hydrolysed was reasonably linear over time for 30 min, and that at the 10 min time point, the activity was completely blocked by 1 µM URB597. In consequence, this time point and cell density were used in the experiments reported in this paper.

### RNA extraction and cDNA synthesis

AT1, C6, RBL-2H3 and P19 cells were plated at a density of 0.7×10^6^ cells per well in 6-well plates. After overnight culturing, cells were washed twice in PBS and total RNA was extracted using the miRNeasy Kit (Qiagen, Hilden, Germany, Cat. No. 217004) according to the manufacturer's instructions. Rat prefrontal cortex was lysed using a homogenisator and RNA extracted as described above. The RNA concentration was measured using a NanoDrop ND-1000 instrument (Thermo Scientific, Wilmington, DE, USA). cDNA was synthesized from 2 µg of total RNA using a cDNA synthesis kit with random primers (High capacity cDNA reverse transcription kit; Applied Biosystems, Foster City, CA, USA). Prior to PCR analysis, cDNA from four wells were pooled for each cell line.

### Semi-quantitative PCR

For detection of rat and mouse FAAH and FABP5 mRNA, cDNA was amplified in polymerase chain reaction (PCR) using a Taq PCR Core kit (Qiagen, Hilden, Germany, Cat. No. 201223), a Biometra TProfessional Gradient 96 thermocycler (Biometra GmbH, Göttingen, Germany) and the following primers: rat FAAH forward 5′-GTGCTGAGCGAAGTGTGGACC-3′, reverse 5′-GGGCCTGGGACAGCTGAGTCT-3′, rat FABP5 forward 5′- CGACCGTGTTTTCTTGCACC-3′, reverse 5′-TGGCATTGTTCATGACGCAC-3′, mouse FAAH forward 5′-GTGGTGCTAACCCCCATGCTGG-3′, reverse 5′-TCCACCTCCCGCATGAACCGCAGACA-3′, mouse FABP5 forward 5′-GACGGTCTGCACCTTCCAAG-3′, reverse 5′-CAGGATGACGAGGAAGCCC-3′. The conditions for PCR were: an initial denaturation step at 94°C for 2 min, followed by 35 cycles with denaturing at 94°C for 40 s, annealing at 60°C for 40 s and elongation at 72°C for 60 s, followed by a final elongation step at 72°C for 3 min. The PCR products were electrophoretically separated on a 1.5% agarose gel, with expected fragment sizes for rat FAAH of 382 bp, rat FABP5 192 bp, mouse FAAH 302 bp, and mouse FABP5 176 bp.

### Statistics

Statistical tests (one- or two-way ANOVA for repeated measures with Dunnett’s or Šídák’s post-hoc multiple tests, as appropriate) were undertaken using GraphPad Prism 5 and 6 for the Macintosh (GraphPad Software Inc., San Diego, CA, USA). The linear regression and Likelyhood ratio analyses of the data with compound 33 were undertaken using the R computer programme [26].

## Results

### Expression and activity of FAAH in AT1, C6, RBL-2H3 and P19 cells

**Figure 1:**
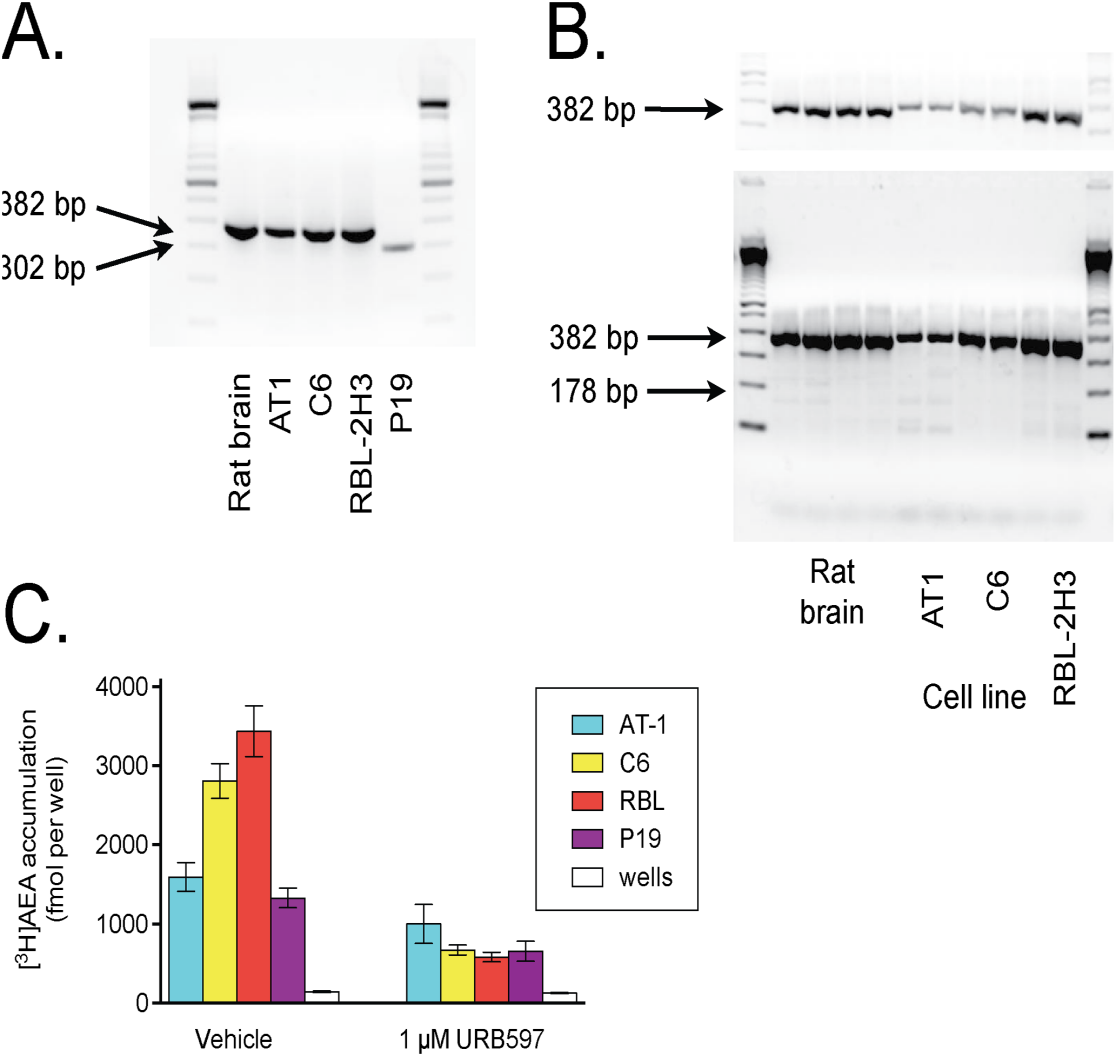
Expression of FAAH and sensitivity of AEA uptake to the FAAH inhibitor URB597 in AT1, C6, RBL-2H3 and P19 cells. In Panels A and B semi-quantitative PCR analysis of the mRNA expression of FAAH are shown for the cells, with rat brain lysates (two different lysates, lanes 2–5, in Panel B) as positive controls. Molecular size markers are also shown. Total RNA was isolated and reverse transcribed into cDNA. Samples were pooled (n = 4) and the PCR analysis was performed using primers designed to recognize rat or mouse FAAH, as appropriate. The PCR products were analyzed by agarose gel electrophoresis and fragment size estimated using a 100 bp marker. The arrows show the expected sizes for FAAH (both Panels) and FLAT (Panel B) with the primer pair used. In Panel B, the main gel shows an overexposure of the gels, with the small panel above showing the band corresponding to FAAH at normal levels of exposure. The photographs have been inverted to show the minor bands. In Panel C, the effect of 1 µM URB597 upon the uptake of [^3^H]AEA is shown for AT1, C6, RBL2H3 and P19 cells. Cells (or wells alone) were preincubated with URB597 for 10 min 37°C followed by addition of 100 nM [^3^H]AEA and incubation for further 4 min at 37°C. Shown are means and s.e.m., n = 5. The statistical treatment of the data is presented in Results.

Semi-quantitative PCR using primers amplifying a PCR product corresponding to the +4 to +385 region of the coding sequence of the rat *faah* gene was performed in order to investigate the expression of FAAH in the three rat cell lines used. As shown in Fig. 1A, the PCR analysis displayed the presence of a band correlating well with the expected fragment size of 382 bp for FAAH mRNA, in all three cell lines and, as a positive control, in rat brain. The expected fragment size of mouse FAAH is 302 bp, and the murine P19 cells expressed a band consistent with this size. In a second experiment (Fig. 1B), the rat cells were run in order to see if a band corresponding to FLAT could be visualised. The top part of the figure shows FAAH expression under conditions of normal exposure. The relative FAAH expression in the samples was AT1<C6<RBL-2H3 cells. No band at 178 bp (the expected size of FLAT as visualised by this primer pair) was seen for either cells or rat brain; however, a weak band (among other bands) was seen at the appropriate size for the rat brain and AT1 cells when the gels were greatly overexposed (main gel in Fig. 1B). The lack of a band corresponding to FLAT under normal exposures is consistent with a recent study in this Journal using mouse tissues and a different primer pair [7]. FAAH activities were also measured in intact cells as part of the experiments described in section 3.3. After incubation of cells (10^5^ per well incubated overnight prior to the experiment) for 10 min with 100 nM added [^3^H]AEA, C6, RBL-2H3 and AT1 cells the tritium recovered in the aqueous phase per well was 3.3±0.66, 2.1±0.16 and 0.31±0.09 pmol, respectively (means ± s.e.m., n = 3). The corresponding value for P19 cells was 0.70±0.09 pmol. Thus, all four cell lines express FAAH, albeit with different activities.

### Inhibition of [^3^H]AEA uptake in AT1, RBL-2H3, C6 and P19 cells by URB597

In Fig. 1C, the uptake of [^3^H]AEA in the absence and presence of 1 µM URB597 is shown for the four cell lines and for wells alone. The cells were assayed concomitantly, and a two-way ANOVA matching for URB597 gave significant effects of cell line (F_3,16_ = 7.96, P<0.005), URB597 (F_1,6_ = 256, P<0.0001) and the interaction term URB597×cell line (F_3,16_ = 32, P<0.0001) [the data for the wells alone were not included]. The significant interaction term allows for *post-hoc* comparisons for vehicle and URB597-treated cells. For the vehicle-treated cells, Šídák’s multiple comparisons test found significant differences between C6 or RBL2H3 cells and AT1 or P19 cells (P<0.001) but not between C6 and RBL2H3 cells or between AT1 and P19 cells. In contrast, for the URB597 treated cells, there were no significant differences between the cell types. The differences in the URB597-sensitive components of uptake between the cells matches well the observed FAAH activity of the intact cells (see above).

**Figure 2:**
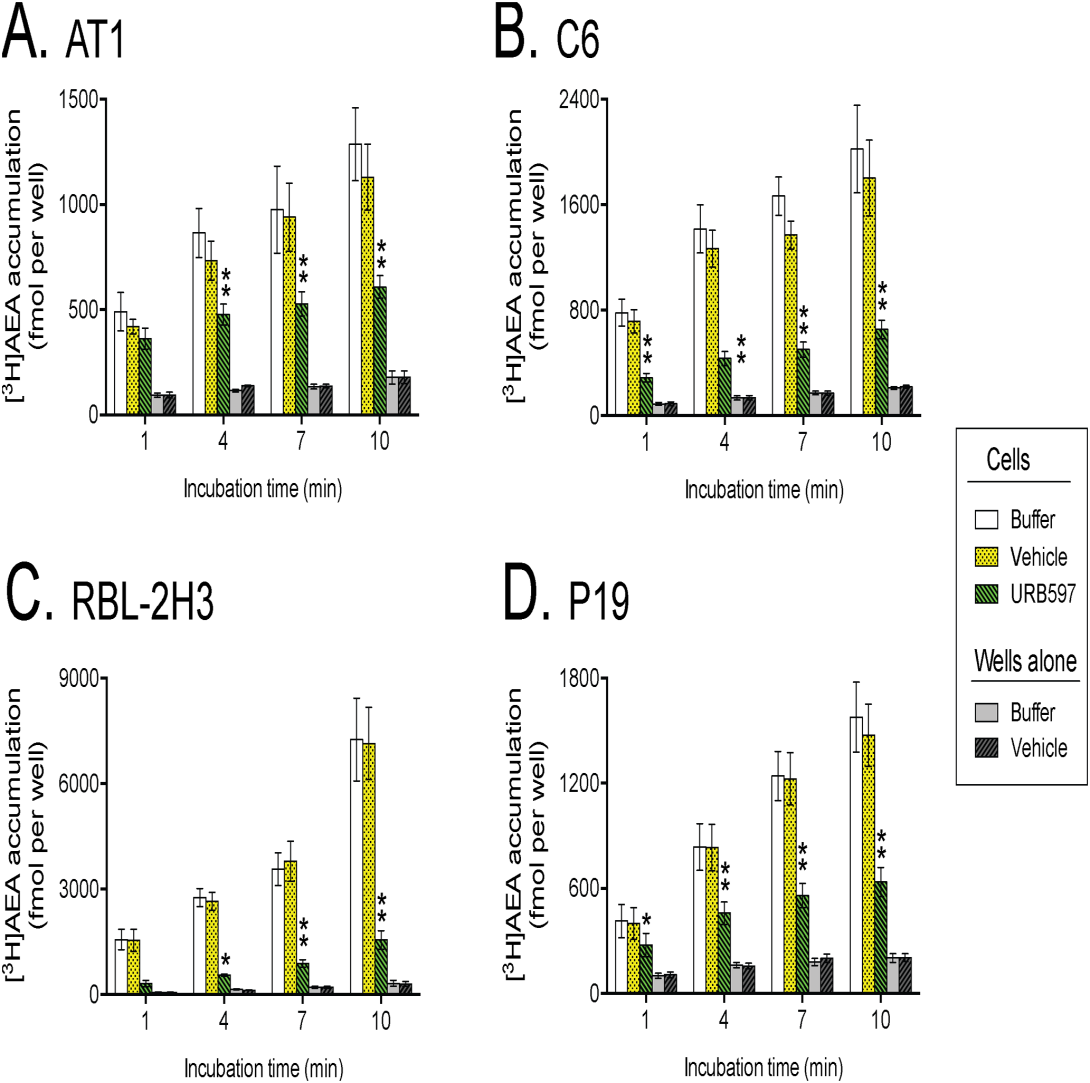
Time-dependent uptake of [^3^H]AEA in AT1, C6, RBL-2H3 and P19 cells treated with either buffer alone, vehicle, or 1 µM URB597. The cells were preincubated for 10[^3^H]AEA. Values are mean and s.e.m. (when not enclosed by the symbols), n = 4 (AT1, C6), 3 (RBL-2H3), or 5 (P19). The concentration of DMSO for the vehicle was 0.2%. For the cell data with vehicle and 1 µM URB597 as variables, two-way repeated-measure ANOVAs matching both time and treatment indicated significant effects of time (P<0.01 for all cells), treatment (P<0.005 for C6, RBL-2H3 and P19; P<0.05 for AT1) and the interaction term time×treatment (P<0.01 for all cells). *P<0.05, **P<0.01, *post-hoc* comparisons using Šídák’s multiple comparison for URB597-treated *vs.* corresponding vehicle-treated cells.

The effect of 1 µM URB597 upon the uptake of AEA into AT1, C6, RBL-2H3 and P19 cells at different incubation times is shown in Fig. 2. For all four cell lines, the rate of uptake was slowed by URB597, as demonstrated by the significant incubation time×treatment interaction term in the two-way repeated ANOVAs (see legend to Fig. 2). Over the period 1–7 min, the rate of uptake for the URB597-treated cells (determined as the slopes of the regression lines for individual experiments) were 30±9, 26±11, 28±5 and 34±4% (means ± s.e.m., n = 3–5) of the corresponding vehicle-treated cells for AT1, C6, RBL-2H3 and P19 cells, respectively.

### The effects of reversible FAAH inhibition upon the cellular uptake and hydrolysis of AEA

**Figure 3:**
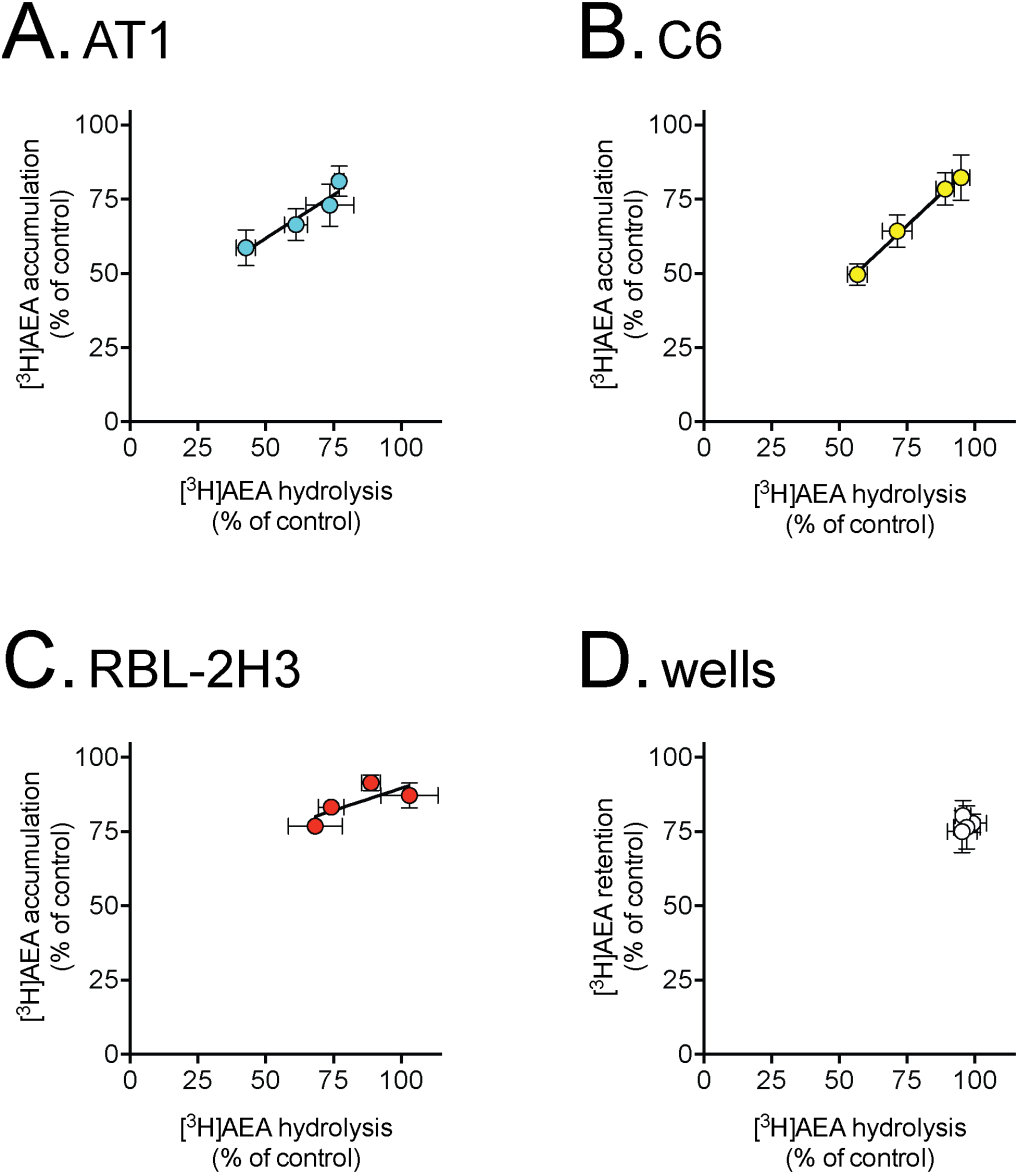
Effect of compound 33 upon AEA uptake and hydrolysis by intact cells. Cells (or wells alone) were pre-incubated with Compound 33, a paracetamol ester with a 2-(4-(2-(trifluoromethyl)pyridin-4-ylamino)phenyl)acetic acid substituent [20], for 10 minutes followed by further incubation with [^3^H]AEA for an additional 4 (uptake; [^3^H-arachidonoyl]-AEA) or 10 (hydrolysis, [^3^H-ethanolamine]-AEA) minutes. The longer time used for the hydrolysis measurements was to allow a sufficient assay: blank ratio. The lack of time-dependency of the inhibition of FAAH by compound 33 [20] permits this difference in incubation times. Shown are means ± s.e.m., n = 3–4. The concentration of EtOH for the vehicle was 0.2% (cells). The values (± s.e.m.) of the slopes determined from the regression lines of the pooled data for each cell were: AT1, 0.58±0.21; C6, 0.85±0.18; RBL-2H3, 0.30±0.12.

Compound 33 of [20] is an inhibitor of FAAH without time-dependence, a property which is advantageous when investigating the effects of FAAH inhibition upon uptake in different assays. We used this compound to compare its ability to inhibit AEA hydrolysis in intact AT1, C6 and RBL-2H3 cells with its ability to affect AEA uptake the same cells. A clear relationship was found between the % reduction of AEA hydrolysis and uptake in all three cells, but not the wells (Fig. 3). From the mean data shown in Fig. 3, linear regression analyses were conducted for three different models in all cases with the mean % FAAH inhibition as an explanatory variable for the mean % uptake inhibition: I, where the intercepts and slopes are the same regardless of cell type. II; where the intercepts are different for the cells but the slopes are not; and III, where there is an interaction term between mean % FAAH inhibition and cell type, thus allowing variation of both slopes and intercepts to occur between cell types. Thereafter, the models were compared using a Likelyhood ratio test. According to this test, model II was preferable to model I, and model III was preferred to model II (P<0.005 in both cases, data not shown). In other words, with the proviso that the standard assumptions about normality of data are fulfilled, the sensitivity of uptake to a given degree of FAAH inhibition, is different between the cells. Thus, although RBL-2H3 and C6 cells express similar mRNA levels of FAAH (Fig. 1), the slope for RBL-2H3 cells is flatter than seen for C6 cells. The slope for the AT1 cells, which express much lower mRNA levels of FAAH than the other two rat cell lines (Fig. 1), is approximately mid way between the slopes for the other two cells.

**Figure 4:**
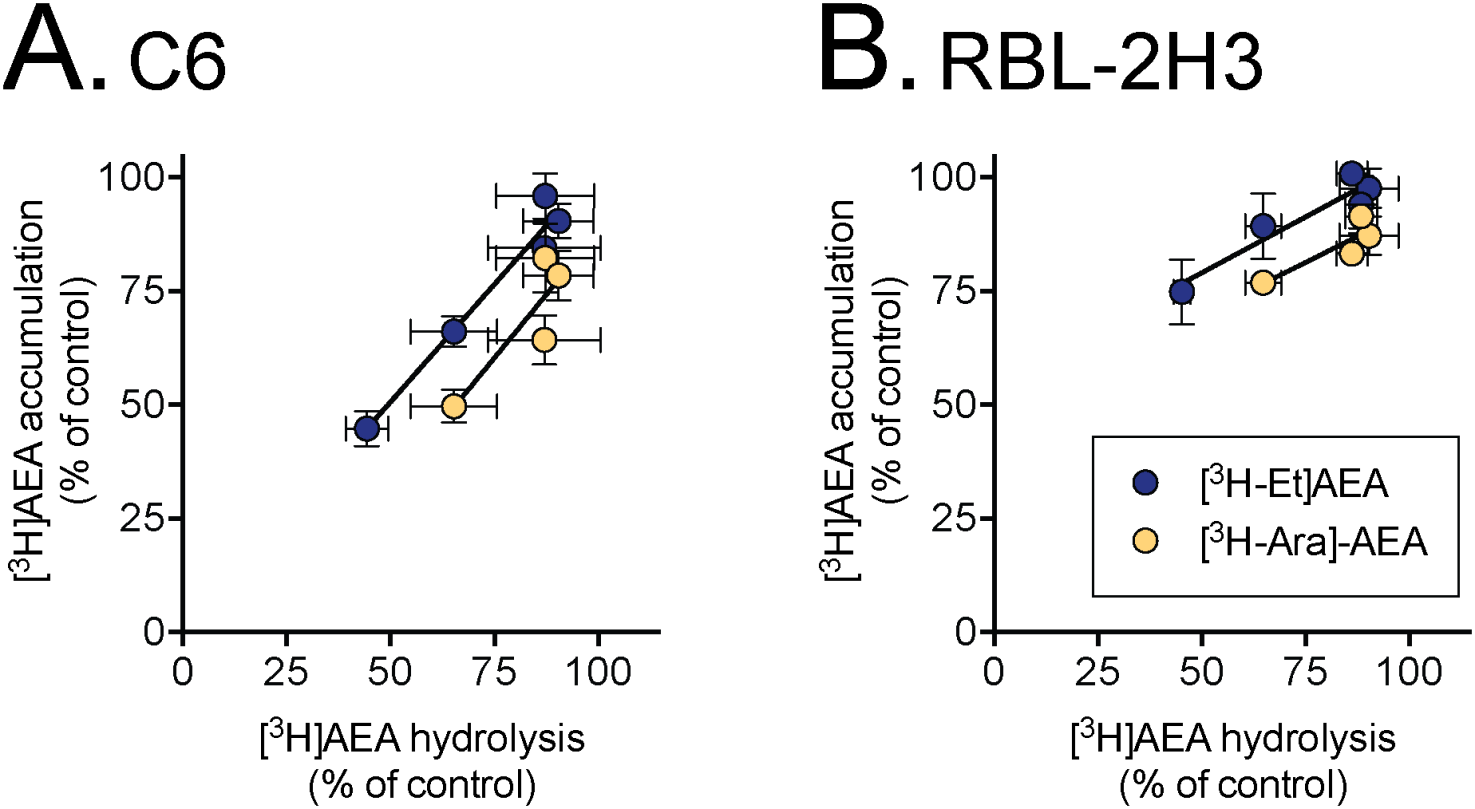
Effect of compound 33 upon AEA uptake using either [^3^H-ethanolamine]- or [^3^H-arachidonoyl]- labelled ligand and hydrolysis of [^3^H-ethanolamine]-AEA by cell lysates. The same conditions as in figure 2 were used. Shown are means ± s.e.m., n = 3–4. The data for [^3^H-arachidonoyl]- labelled AEA uptake is the same as for Fig. 2, but in this case the x-axes are for cell lysates rather than intact cells. The values (± s.e.m.) of the slopes determined from the regression lines of the pooled data for [^3^H-ethanolamine]- or [^3^H-arachidonoyl]- labelled AEA, respectively, were: C6 cells,, 1.03±0.10 and 1.11±0.31; RBL-2H3 cells, 0.49±0.14 and 0.46±0.14. The concentration of EtOH for the vehicle was 0.2% (cells) and 1% (lysates).

It can be argued that a comparison between AEA labelled in the arachidonoyl part of the molecule (for uptake) *vs.* AEA labelled in the ethanolamine part of the molecule (for FAAH) may be misleading given that the FAAH reaction products will have different subsequent metabolic fates in the cells. Further, the use of different incubation times (4 min for uptake vs. 10 min for hydrolysis) may be a complicating factor, although compound 33 was chosen because of its lack of time-dependency. In consequence, we investigated the effects of compound 33 upon the uptake of [^3^H]AEA labelled in the ethanolamine part of the molecule by C6 and RBL2H3 cells. Further, we investigated the effects of this compound upon the AEA hydrolytic activity of C6 and RBL2H3 cell lysates (Fig. 4). Compound 33 produced essentially identical levels of FAAH inhibition in the lysates of the two cell types, but the difference in slopes between the cell types seen for the intact cells was retained. Further, the slopes for the regression lines were the same (for a given cell) for the [^3^H]AEA labelled in the ethanolamine part as for the [^3^H]AEA labelled in the arachidonoyl part of the molecule (see legend to Fig. 4).

### Expression of FABP5 by AT1, RBL-2H3, C6 and P19 cells

**Figure 5:**
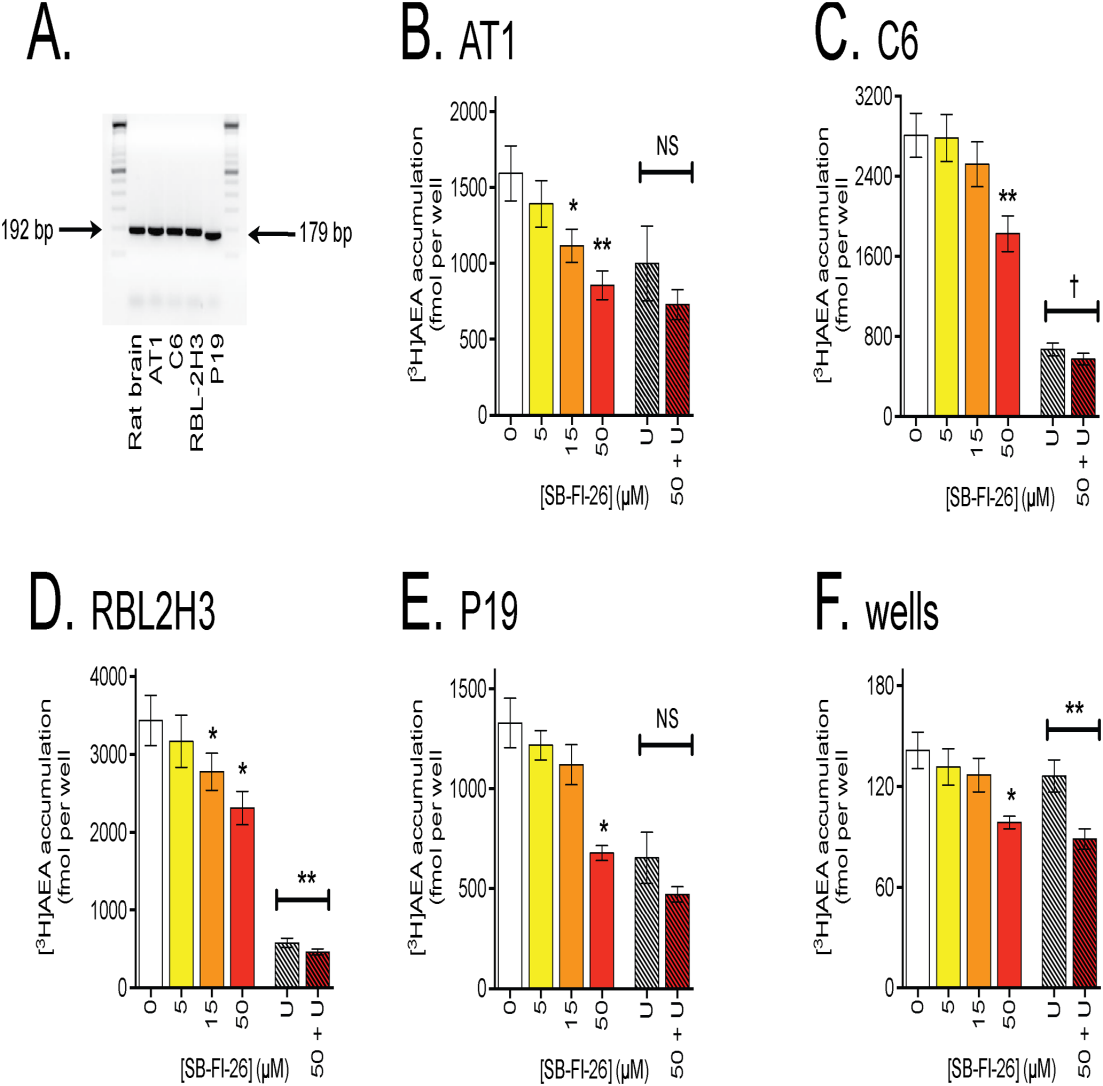
FABP5 expression and function in the cells. In Panel A, semi-quantitative PCR analysis of the mRNA expression of FABP5 is shown for the cells, with a rat brain lysate as positive control. Molecular size markers are also shown. Panels B-E show the effect of SB-FI-26 and 1 µM URB597 (“U”) upon the accumulation of [^3^H]AEA in AT1, C6, RBL2H3, P19 cells and upon the retention of label by wells alone. Note that the scales on the y-axis are different, particularly for the wells, where the rate of accumulation is very low. *P<0.05, **P<0.01 either Dunnett’s multiple comparisons test vs. vehicle control following significant one way repeated measures ANOVA not assuming sphericity (for SB-FI-26 concentration-response data) or paired t-test between 50 µM SB-FI-26+1 µM URB597 vs. URB597 alone (NS, not significant; †, P = 0.054). All values are means ± s.e.m., n = 5. Note that the vehicle control and 1 µM URB597 data are the same as shown in Fig. 1C.

FABP5 is an important intracellular carrier of AEA [4], raising the possibility that the differential effects seen between C6 and RBL-2H3 cells in Figs. 3 and 4 may reflect differences in expression levels of this protein. In consequence, we investigated the mRNA expression levels and function of FABP5 in the cells, the latter using the FABP5 inhibitor SB-FI-26 [27]. The PCR analysis displayed the presence of a band correlating well with the expected fragment size of 192 bp for rat FABP5 mRNA (rat brain, AT1, C6, RBL-2H3) and 179 bp for mouse FABP5 mRNA (P19 cells). Further, the expression levels were very similar for the different cells (Fig. 5A). The effects of 5, 15 and 50 µM SB-FI-26 upon the uptake of AEA by the cells, and upon the retention of AEA by the wells alone, are shown in Figs. 5B–F (note that the scales on the y-axes are very different for the cells and for the wells). In all cases 50 µM SB-FI-26 reduced the observed uptake, but it even affected the rate of AEA retention by the wells, which complicates interpretation of the data. In the RBL2H3 cells (and wells), the combination of 50 µM SB-FI-26 and 1 µM URB597 produced a greater reduction in uptake than seen with URB597 alone, whereas in the other cells no significant differences were seen at the P<0.05 level.

**Figure 6:**
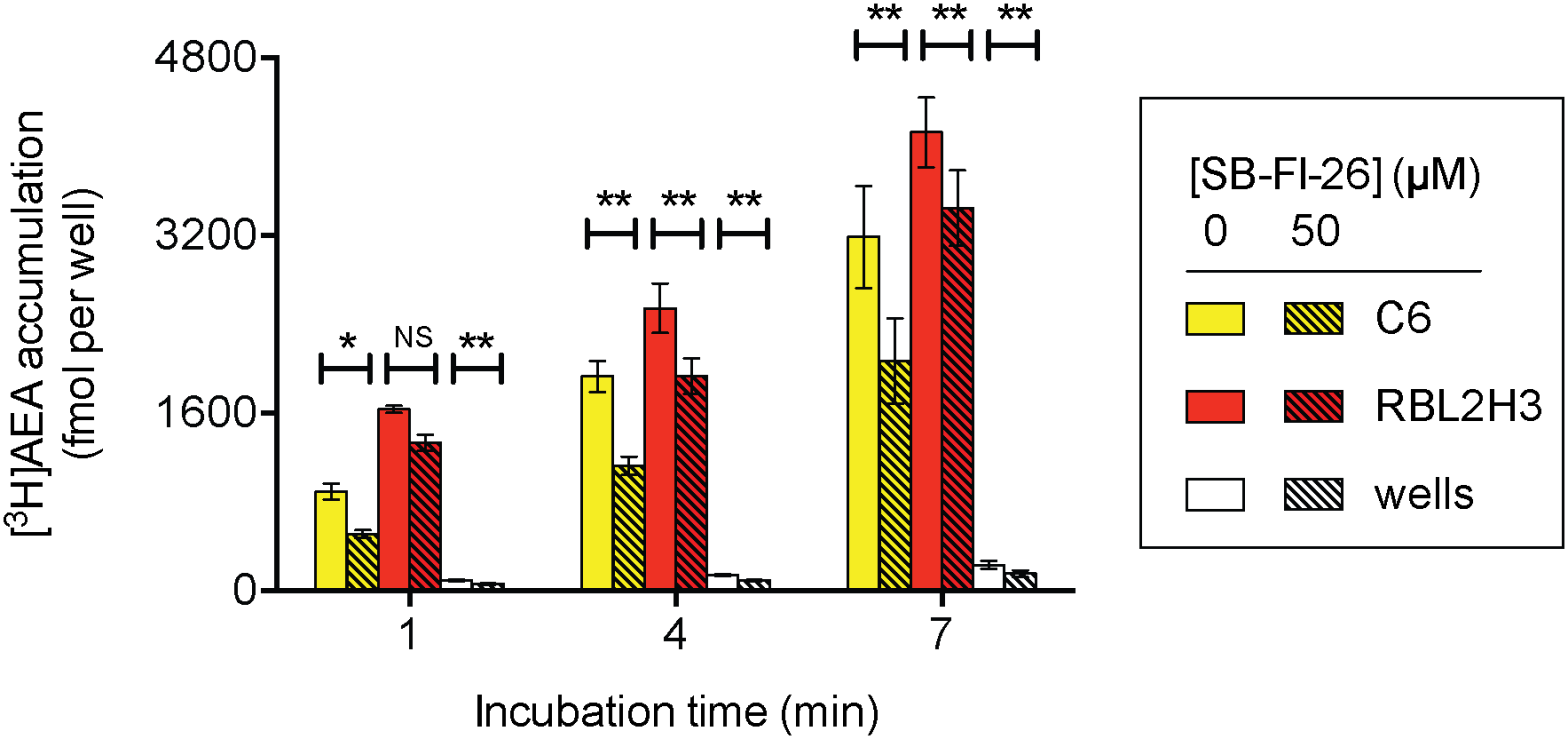
Inhibition of AEA uptake in C6 and RBL-2H3 cells by SB-FI-26 at different incubation times. Shown are means and s.e.m., n = 5, for the uptakes measured at each time point. Two-way ANOVA matching for both time and SB-FI-26 were undertaken separately for each cell line and for the wells. **P<0.01, *P<0.05, Šídák’s multiple comparison test. Note that for the RBL-2H3 cells, the interaction term time×SB-FI-26 did not reach significance, and so the difference in significance at the different time points should not be over-interpreted. For both cell lines and for the wells, the ANOVA values were significant (P<0.005) for the main effects of time and of SB-FI-26 treatment.

The effect of 50 µM SB-FI-26 upon the adsorption to the wells is small in absolute terms, and can be further minimised by following the change in uptake/adsorbtion over time. These data are shown with C6 and RBL-2H3 cells and in wells in Fig. 6. Assuming linearity over the 1–7 min incubation times, the rates of uptake (in fmol/min per well) in the absence and presence of 50 µM SB-FI-26, respectively, calculated from the individual experiments were, C6 cells, 382±70 and 260±95; RBL-2H3, 416±58 and 352±54; wells, 23±6 and 15±4. A two-way repeated measures ANOVA of the data for the C6 and RBL-2H3 cells matching for SB-FI-26 indicated a significant effect of the compound (F_1,8_ = 22, P<0.005), but not for cell (F_1,8_ = 2.22, P = 0.17) or the interaction term cell×SB-FI-26 (F_1,8_ = 0.57, P = 0.47). Thus, these data do not support the hypothesis that differences in FABP-5 expression or function lie behind the different sensitivities of AEA uptake by C6 and RBL-2H3 cells to reversible inhibition of FAAH.

## Discussion

Although the metabolic pathways for AEA are well characterized, the mechanisms responsible for the cellular uptake of AEA still remain to be completely explained (review, see [3]). One point of contention is the degree to which FAAH regulates the uptake of AEA. In the present study, we have investigated this in some detail using cells with different expression levels of the enzyme. Two approaches were taken: in the first, time courses of the reduction of the rate of uptake following irreversible FAAH inhibition were investigated. For all three cell lines, 1 µM URB597 (which completely inhibits hydrolysis in the C6 cells) produced a 70% reduction in the rates of uptake between 1 and 7 min incubations. It is notable that for the C6 cells, a significant effect of URB597 is seen even at the 1 minute incubation time. This would suggest that sufficient AEA has reached FAAH for it to be metabolised by 1 min of incubation; thereby at this time point, the enzyme contributes to the intra: -extracellular concentration gradient. This is reinforced by our finding that the FABP-5 inhibitor SB-FI-26 also produces a significant reduction in uptake at the 1 min time point, assuming, of course, that FABP5 plays a part in the shuttling of AEA to FAAH in this cell line. More conclusively, it has been shown that in RBL-2H3 and U937 human monocytic leukaemia cells, AEA hydrolysis can be demonstrated at very short incubation times (25–30 sec) [12], [19], and so this explanation seems feasible.

A more surprising finding, however, was the difference in the sensitivies of uptake to modest levels of reversible inhibition. The basic finding, that there was a relationship between % inhibition of AEA hydrolysis and uptake is consistent with the study of Kaczocha et al. [19]. These authors compared the % uptake and % hydrolysis using both FAAH inhibitors (three concentrations of CAY10400, two concentrations of URB597) and two concentrations each of uptake inhibitors (OMDM2, AM1172, VDM11 and UCM707), and found a linear relationship for the complete dataset [19]. However, in our study, RBL-2H3 cells were less sensitive than both the C6 cells (which had broadly similar FAAH expression) and the AT1 cells (which have a much lower FAAH expression) to a given level of FAAH inhibition by compound 33. Such a difference indicates that the reported variation in FAAH sensitivities of AEA uptake seen by different authors (see Introduction) need not be ascribed to methodological differences, but instead can reflect the properties of the cells themselves. Given than the maximal inhibition of the rate of uptake seen with 1 µM URB597 between 1 and 7 min is the same in percentage terms for all four cells, the data would suggest that in RBL-2H3 cells, the effect of inhibition of FAAH upon uptake “catches up” following the lag in sensitivity between 0 and 50% inhibition. The difference in sensitivities between the cell lines, in particular between RBL-2H3 and C6 cells, is unlikely to be due to differences in FABP5 involvement, given the similar inhibition profiles with SB-FI-26 in the cells, but this of course does not rule out cell-dependent differences in the involvement of other AEA shuttling pathways. The mechanism(s) by which intracellular AEA is transported to its catabolic and/or sequestration sites is not fully understood, although several molecular targets (in addition to FABP-5) and sequestration sites have been reported, and may be amenable to pharmacological attack [5], [28], [29]. As argued by Hillard and Jarrahian [30] cells may accumulate AEA for different reasons depending upon whether this lipid is required for signalling purposes or as a source of arachidonic acid. Although variations in the intracellular fate of AEA presumably explain the subtle differences seen in the present study.
